# Surgical treatment of a symptomatic os acromiale by arthroscopy-assisted double-button fixation: a case report

**DOI:** 10.1007/s00402-022-04341-4

**Published:** 2022-01-21

**Authors:** Larissa Eckl, Markus Scheibel

**Affiliations:** 1 Department of Shoulder and Elbow Surgery, Schulthess Clinic, Zurich, Switzerland; 2 Department of Shoulder and Elbow Surgery, Center for Musculoskeletal Surgery, Charité-Universitaetsmedizin Berlin, Augustenburger Platz 1, 13353 Berlin, Germany

**Keywords:** Acromion, Double-button fixation, Case report, Arthroscopy, Shoulder

## Abstract

**Case:**

We present the case of a symptomatic os acromiale in a 51-year-old female patient. Arthroscopy-assisted treatment was performed using a double-button fixation system and additional suture cerclage. The patient presented with complete radiographic bone union, pain relief, improved range of motion and did not require hardware removal at the 12-month follow-up.

**Conclusion:**

The achievement of persistent consolidation between the two fragmented bone surfaces, without further need for hardware removal and improved clinical outcome, suggests that our minimally invasive technique is appropriate for this specific indication. To our knowledge, this technique has not been described in the literature yet.

## Introduction

An os acromiale occurs as an unfused part of the acromion, resulting from a lack of ossification between the three final ossification centers of the acromion (preacromion, mesacromion and metacromion) [1–6]. This process of bone formation is usually completed by around 25 years of age [1, 5, 7–10]. Nevertheless, the reported incidence of deficient osseous fusion, leading to a disunited fragment ranged from 1 to 30% [2, 4, 5, 8, 10–12].

In the majority of unfused cases, an os acromiale is asymptomatic and incidentally detected on shoulder radiographs [6–8, 11, 13]. Alternately, painful conditions including tenderness on palpation, complaints during overhead activities, pain due to sleeping on the affected shoulder, positive signs of impingement and weakness of the rotator cuff muscles [6, 7, 10–12] frequently occur after trauma or may have atraumatic origins [4, 6–8]. Symptomatic patients should initially receive conservative management involving physiotherapy, analgesia and supplementary corticoid injections for at least 6 months [2, 6–9, 11, 12]. To alleviate persistent complaints after failed nonoperative treatment, numerous surgical procedures, such as open or arthroscopic fragment excision, open or arthroscopic acromioplasty, several techniques of open reduction and internal fixation and arthroscopy-assisted open reduction and internal fixation have been described in the literature [2, 6, 7, 9, 10]. Unfavorable clinical and radiographic outcomes related to these interventions have been attributed to hardware migration, the need for postsurgical hardware removal, persistent pain and radiographic nonunion [6, 7, 9–11]. We present the case of a symptomatic patient who was treated by arthroscopy-assisted fixation using a double-button fixation system and additional suture cerclage to achieve sufficient compression and stabilization of the unfused bone.

The patient provided written consent after being informed that data concerning her case would be submitted for publication.

## Case report

A 51-year-old female patient in a good general state of health presented to our shoulder department because of recurrent pain of the left shoulder, after reported trauma 2 years ago where she fell on her outstretched arm and sustained a wrist fracture.

**Fig. 1:**
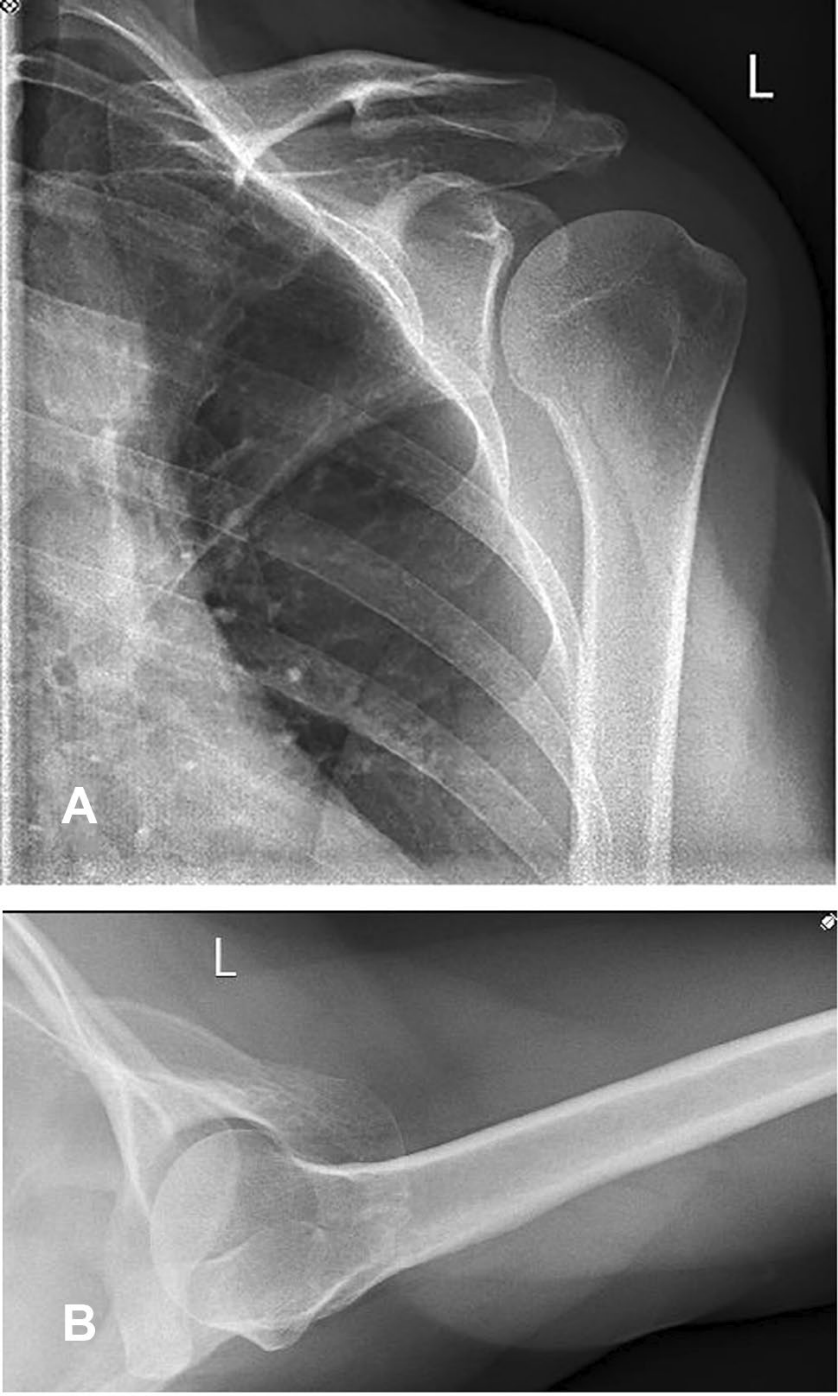
**A**–**B** Preoperative anteroposterior and axillary view radiographs

**Fig. 2:**
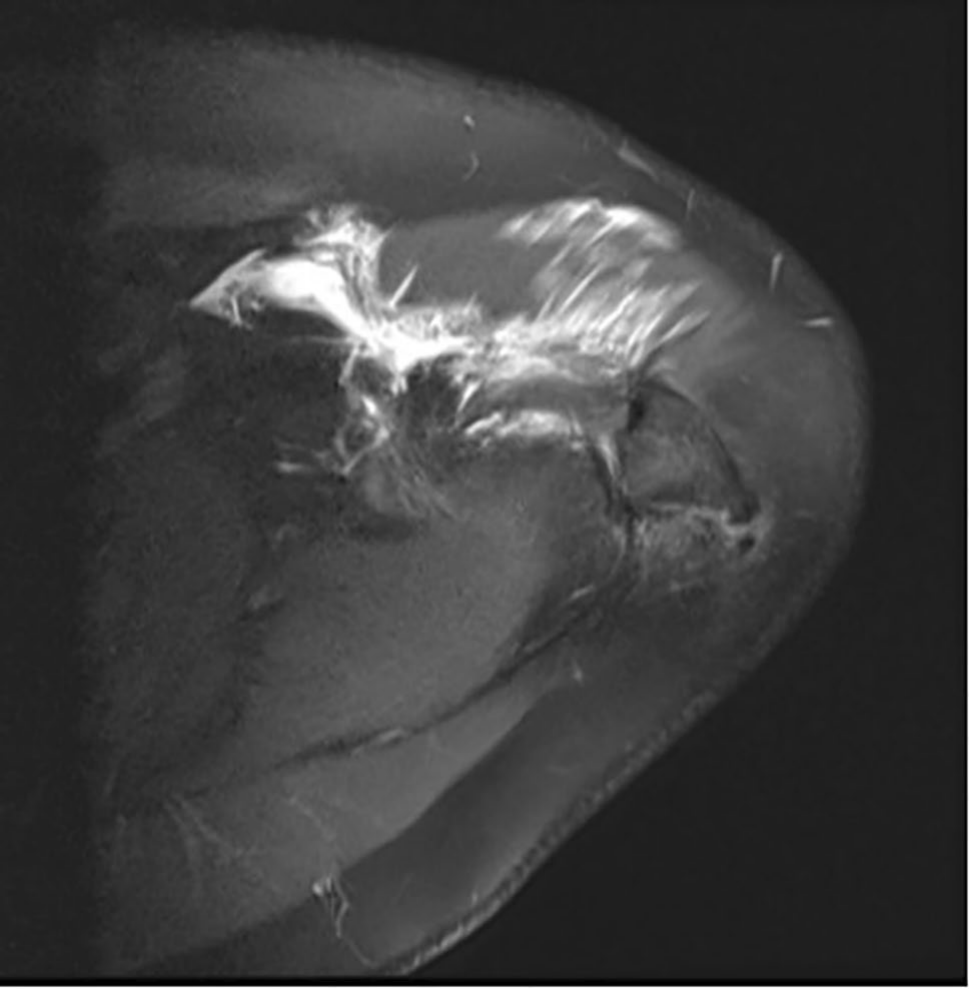
Magnetic resonance image depicting the os acromiale

Clinical examination revealed tenderness and pain above the acromial region with a positive impingement sign according to Neer [14]. Active and passive range of motion could be performed without any restrictions. Conventional radiographs and magnetic resonance imaging depicted an os acromiale indicated by a 2.5 cm long fragment in connection with the acromioclavicular joint and additional inflammation of the subacromial bursa (Figs. 1 and 2).

Due to failed conservative management, including local corticoid injections for more than 6 months, the patient consented to undergo surgery.

Under general anesthesia, the patient was placed in a beach chair position and the affected left shoulder was sterilely prepared. A standard diagnostic arthroscopy was performed via a posterior viewing portal and no intraarticular lesions were detected.

**Fig. 3:**
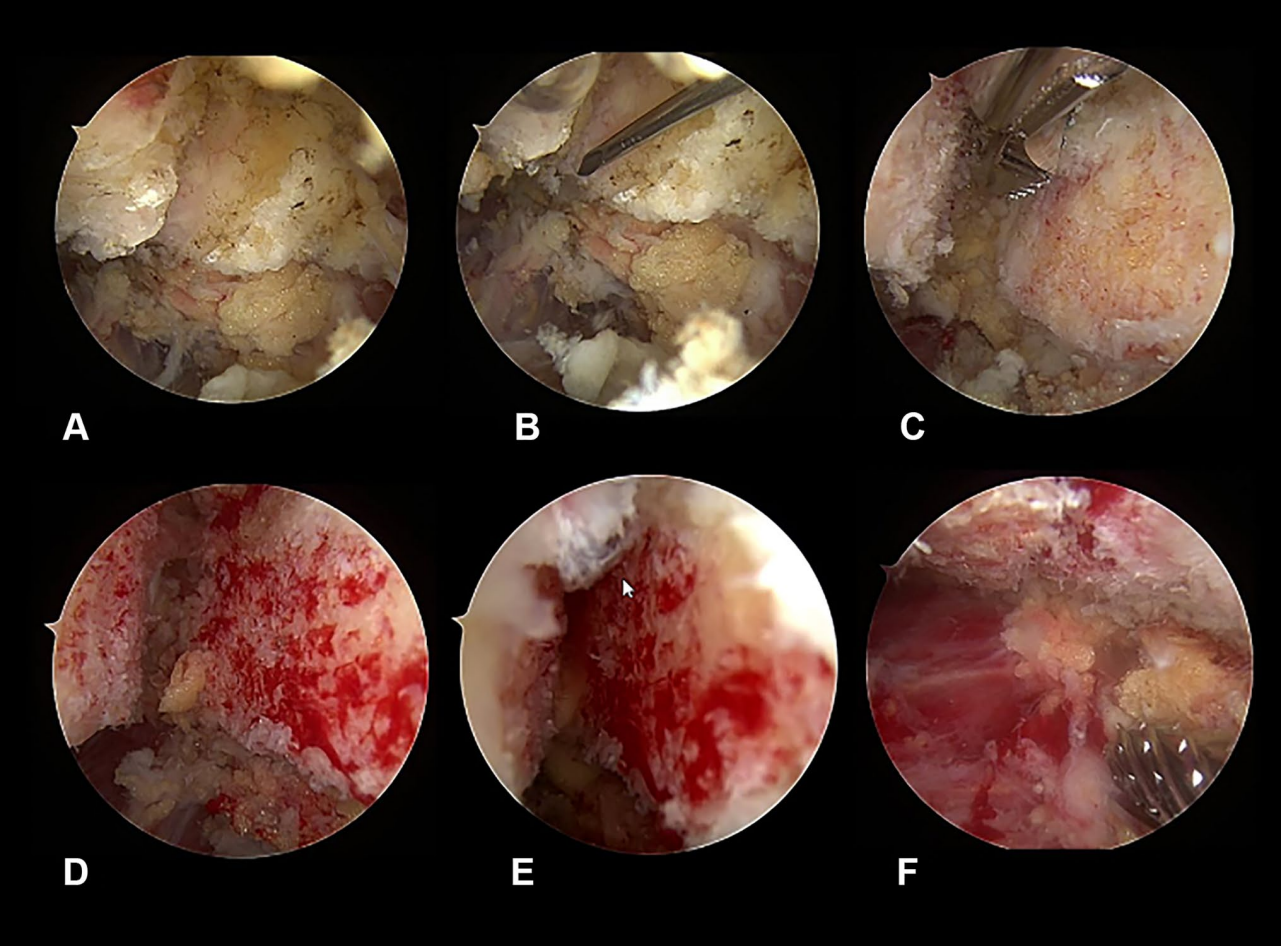
**A**–**F** Arthroscopic views showing the osseous gap between the two bone surfaces of the os acromiale (**A**–**C**), preparation of the bone surfaces (**D**), process of closing the gap (**E**) and complete consolidation of the two bone surfaces (**F**)

Hereafter, the subacromial bursitis was verified followed by a partial bursectomy. The nonunion side of the os acromiale was confirmed and the two fragmented surfaces were debrided to achieve optimal healing conditions (Fig. 3A–C). Two 2.0 mm k-wires were placed parallel from posterior to anterior through the acromion close to the osseous gap under radiographic control. A bone reduction forceps was applied to accomplish high compression to completely close the separation (Fig. 3D–F). The k-wires were finally advanced into the anterior fragment.

Since the osseous structure of the os acromiale turned out fairly soft and may compromise the stability of screw fixation, we decided upon cortical stabilization instead of a transosseous compression, using a double-button fixation system. Guided by the lateral k-wire, the posterior cortex of the acromion was monocortically overdrilled with a 5.1 mm drill to place the pilot hole for insertion of the low-profile button. A 3.5 mm cannulated drill bit was used to complete tunnel placement for the low-profile TightRope® device (Arthrex, Naples, FL). Guided by a nitinol wire that was placed via the cannulated drill bit, the sutures of the TightRope® device were pulled through the acromion in an anterior direction. A Dog Bone™ button (Arthrex, Naples, FL) was placed on the ventral surface of the acromial edge and shuttled in reverse into its final position. The top head button was adjusted in the posterior part of the drill hole by tensioning the free suture limbs. High compression of the two fragmented surfaces was accomplished using a suture tensioner with 80–100 N. To ensure a maximum of consolidation, an additional suture cerclage was attached to provide backup fixation. Therefore, the second 2.0 mm k-wire was exchanged by a 1.25 mm k-wire and overdrilled with a 2.7 mm cannulated drill. A replacing nitinol wire attached with the FiberTape® (Arthrex, Naples, FL) was pulled through the acromion and tightened by the suture tensioner after being subcutaneously shuttled backwards. The sutures were knotted and shortened at the level of the low-profile button to avoid knot stack.

**Fig. 4:**
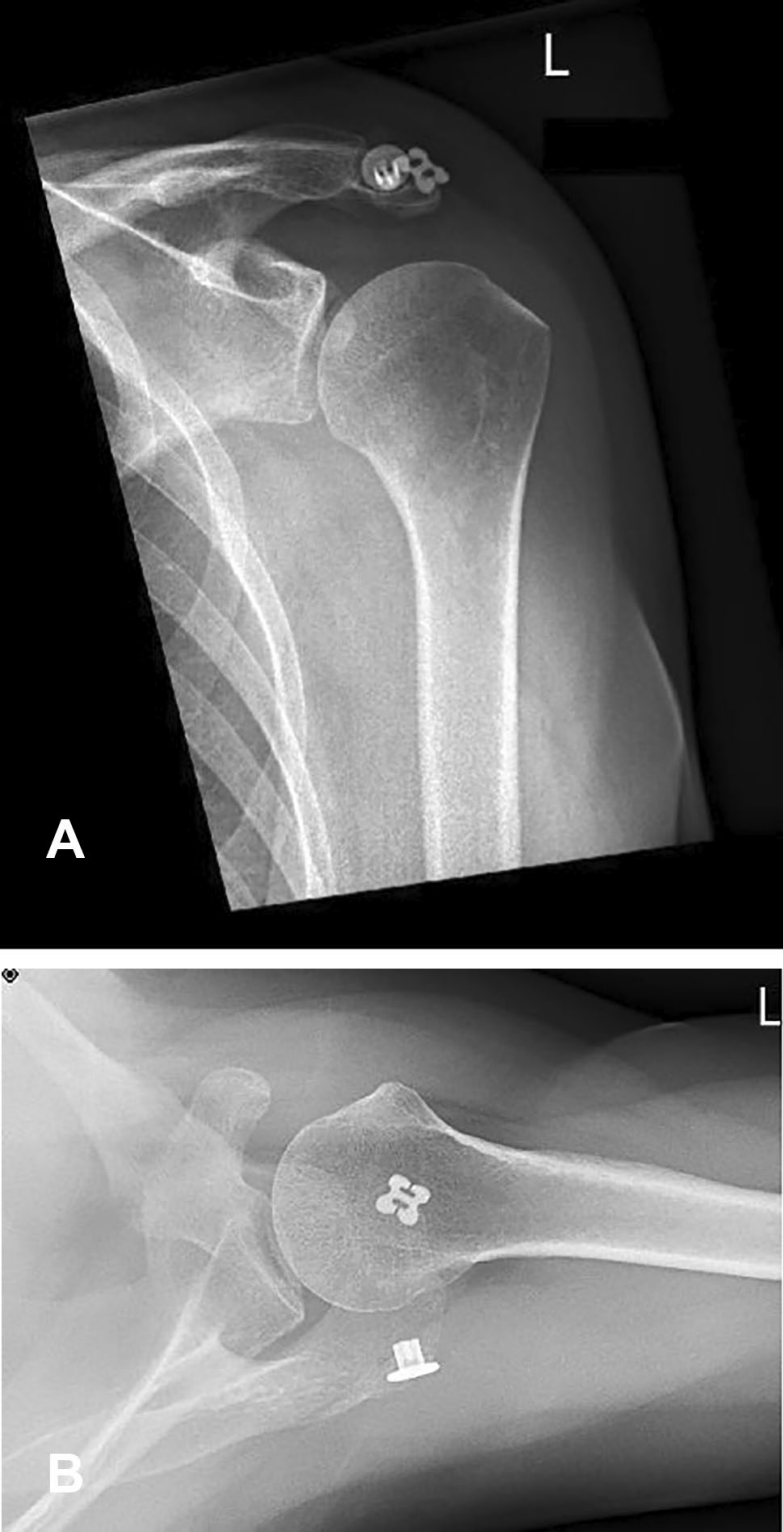
**A**–**B** Anteroposterior and axillary view radiographs at the 12-month follow-up

**Fig. 5:**
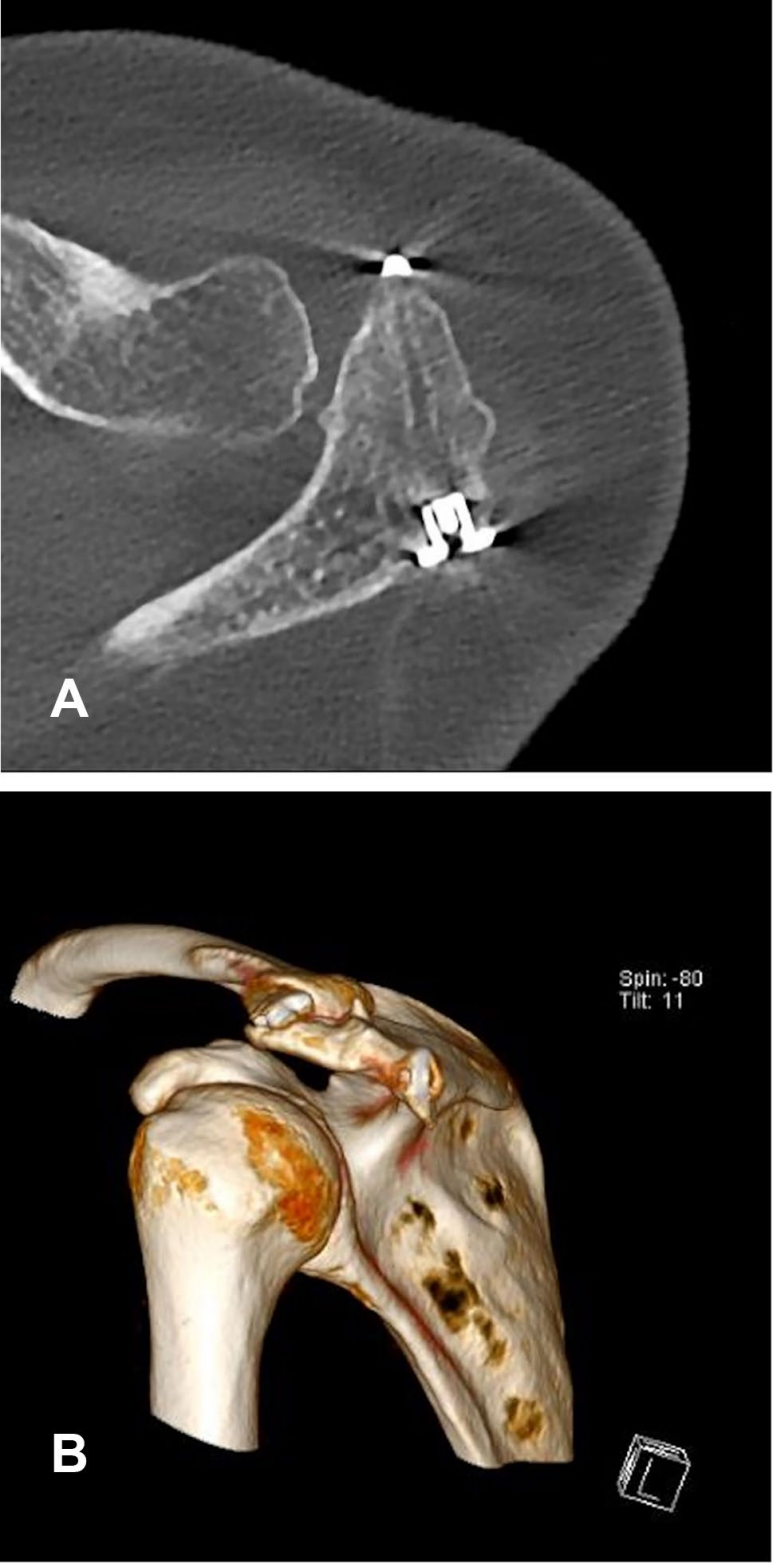
**A**–**B** Computer tomography control scan at the 12-month follow-up showing persisting consolidation of the os acromiale and good implant position

Postoperative rehabilitation involved initial shoulder immobilization in a Donjoy® Ultrasling® (DJO Global) for 8 weeks accompanied by physiotherapy and analgesia. One year after surgery, the persisting consolidation of the os acromiale and the proper position of the implant were confirmed by radiographs and computer tomography imaging (Figs. 4 and 5). The patient remained pain free and the hardware was not palpable. No noticeable discomfort around the implants was experienced. The assessment of the subjective shoulder value (SSV) showed 90%. The shoulder range of motion improved to 180° flexion, 170° abduction and 50° external rotation.

## Discussion

Clinical and radiological outcome improve after surgical treatment of a symptomatic os acromiale following failed conservative management [7, 10]. In particular, arthroscopic procedures are intended to preserve the deltoid muscle and fascia, to prevent damage of the blood supply, to improve the cosmetic outcome and to provide the option for treatment of associated pathologies [2, 9, 10].

In the case of an osseous gap between the mesacromion and metacromion, as described in our patient, internal fixation achieves adequate radiological and clinical outcome [7, 9]. In a comparison of numerous internal fixation techniques for symptomatic os acromiale, including fixation by screws or k-wires, with or without additional fixation by tension band with either wire or nonabsorbable sutures, Viner et al. reported that the screw technique leads to a higher rate of radiographic union than the k-wire technique [7]. The apparent fusion of the bone fragments, depicted by radiographs, is related to significantly higher clinical outcomes [6, 7, 10].

Despite existing bone union, complaints and pain as a cause of hardware irritation are not uncommon [9, 11, 15]. Abboud et al. achieved a rate of bone union in 100% of the patients treated by open reduction and internal fixation with either figure-of-eight wiring or fixation by cancellous screws; nevertheless, only 38% of the patients had a satisfactory outcome [15]. There was a significant rate of pain by reason of hardware irritation in both, the screw group and the figure-of-eight wiring group, resulting in an overall surgical hardware removal rate of 88% [15]. A more differentiated view showed that the need for hardware removal is more often described after the k-wire technique than fixation by screws [6, 7, 10].

A more recent study by Guo et al. described the treatment of a symptomatic os acromiale by means of arthroscopic fixation with two polyester sutures [12]; this procedure was reasoned to avoid hardware irritation and prevent the risk of acromial bone fractures, because the need for acromial drill holes is eliminated. At the 12-month follow-up, all patients achieved bone union as shown on control computer tomography scans [12]. To ensure persistent prospective compression of the two fragmented bone surfaces and to guarantee even stronger compression, we decided to use a double-button fixation system instead of sutures alone. Since the two buttons are placed into the ventral and dorsal cortex, the construction is not dependent on transosseous compression in case of an insufficient osseous structure. The concern about a possible adverse event of irritation due to hardware migration favored the decision. This specific fixation device is usually used for the fixation of acute acromioclavicular joint instability and reported adequate outcomes of minimal soft tissue damage, no need for hardware removal, excellent cosmetic results and the capability to use a minimal invasive procedure for its application [16–18]. For our patient, primary fixation with the double-button device and additional suture cerclage to support the initial fixation, led to excellent radiographic results and improvement in clinical outcome 1 year after surgery.

As a result of this procedure, tight compression is simultaneously exerted by two different mechanisms that provide mutual stabilization. Although osseous drilling is necessary, there is no need for more drill holes compared to internal fixation by screws or k-wires but in contrast, as the result of our technique the bone union is ensured by a combined double fixation. Hardware removal is an additional important aspect of this procedure that needs no consideration. Due to the profile of the low-profile button no knot stack appears, and the hardware was neither ventral nor dorsal palpable. Our patient did not experience any discomfort or irritation around the implants.

## Conclusion

Arthroscopy-assisted fixation of the symptomatic os acromiale in our patient using a double-button device combined with a suture cerclage showed excellent radiographic results. At the 12-month follow-up, complete consolidation was observed. Due to the minimal invasive technique, a satisfactory cosmetic result was achieved with nominal damage to the surrounding soft tissue. Hardware removal was not required; the patient showed improved range of motion without persistent complaints and the subjective shoulder value was 90%. Therefore, the outlined surgical technique represents a feasible procedure for treating symptomatic os acromiale after failed conservative management. Despite our satisfactory results, a potential discomfort due to hardware irritation and the cost of the implant in comparison to the application of screws or k-wires must be considered. Further work is still necessary, however, to examine whether fragment size or location plays a role in the effectiveness of this treatment technique.

## References

[1] McClure JG, Raney RB (1975). Anomalies of the scapula. Clin Orthop Relat Res.

[2] You T, Frostick S, Zhang W-T, Yin Q (2019). Os acromiale: reviews and current perspectives. Orthop Surg.

[3] Macalister A. Notes on acromion. J Anat Physiol. 1893 Jan;27(Pt 2):244.1–251. PMC1328282 17232028

[4] Sammarco VJ (2000). Os acromiale: frequency, anatomy, and clinical implications. J Bone Joint Surg Am.

[5] Prescher A (2000). Anatomical basics, variations, and degenerative changes of the shoulder joint and shoulder girdle. Eur J Radiol.

[6] Spiegl UJ, Millett PJ, Josten C, Hepp P (2018). Optimal management of symptomatic os acromiale: current perspectives. Orthop Res Rev.

[7] Viner GC, He JK, Brabston EW, Momaya A, Ponce BA (2020). Os acromiale: systematic review of surgical outcomes. J Shoulder Elbow Surg.

[8] Johnston PS, Paxton ES, Gordon V, Kraeutler MJ, Abboud JA, Williams GR (2013). Os acromiale: a review and an introduction of a new surgical technique for management. Orthop Clin North Am.

[9] Kurtz CA, Humble BJ, Rodosky MW, Sekiya JK (2006). Symptomatic os acromiale. J Am Acad Orthop Surg.

[10] Harris JD, Griesser MJ, Jones GL (2011). Systematic review of the surgical treatment for symptomatic os acromiale. Int J Shoulder Surg.

[11] Ortiguera CJ, Buss DD (2002). Surgical management of the symptomatic os acromiale. J Shoulder Elbow Surg.

[12] Guo D-M, Li Z-X, Wang Q, Song H-H (2019). Fixation of os acromiale using polyester sutures: a novel surgical treatment. Ann Transl Med.

[13] Warner JJ, Beim GM, Higgins L (1998). The treatment of symptomatic os acromiale. J Bone Joint Surg Am.

[14] Neer CS (1983). Impingement lesions. Clin Orthop Relat Res.

[15] Abboud JA, Silverberg D, Pepe M, Beredjiklian PK, Iannotti JP, Williams GR, Ramsey ML (2006). Surgical treatment of os acromiale with and without associated rotator cuff tears. J Shoulder Elbow Surg.

[16] Torkaman A, Bagherifard A, Mokhatri T, Haghighi MHS, Monshizadeh S, Taraz H, Hasanvand A (2016). Double-button fixation system for management of acute acromioclavicular joint dislocation. Arch Bone Jt Surg.

[17] Boileau P, Old J, Gastaud O, Brassart N, Roussanne Y (2010). All-arthroscopic Weaver-Dunn-Chuinard procedure with double-button fixation for chronic acromioclavicular joint dislocation. Arthroscopy.

[18] Minkus M, Maziak N, Moroder P, Scheibel M (2019). Arthroscopic low-profile reconstruction for acute acromioclavicular joint instability. Obere Extremität.

